# Population genomics meet Lagrangian simulations: Oceanographic patterns and long larval duration ensure connectivity among *Paracentrotus lividus* populations in the Adriatic and Ionian seas

**DOI:** 10.1002/ece3.2844

**Published:** 2017-03-14

**Authors:** Marta Paterno, Marcello Schiavina, Giorgio Aglieri, Jamila Ben Souissi, Elisa Boscari, Renato Casagrandi, Aurore Chassanite, Mariachiara Chiantore, Leonardo Congiu, Giuseppe Guarnieri, Claudia Kruschel, Vesna Macic, Ilaria A. M. Marino, Chiara Papetti, Tomaso Patarnello, Lorenzo Zane, Paco Melià

**Affiliations:** ^1^ Department of BiologyUniversity of PadovaPadovaItaly; ^2^ Consorzio Nazionale Interuniversitario per le Scienze del Mare (CoNISMa)RomaItaly; ^3^ Dipartimento di Elettronica, Informazione e BioingegneriaPolitecnico di MilanoMilanoItaly; ^4^ Department of Biological and Environmental Sciences and TechnologiesUniversity of SalentoLecceItaly; ^5^ Institut National Agronomique de Tunisie (INAT)TunisTunisia; ^6^ USR 3278 CNRS‐EPHECRIOBEUniversité de Perpignan Via DominitiaPerpignan CedexFrance; ^7^ Department for Earth, Environment and Life Sciences (DiSTAV)University of GenoaGenoaItaly; ^8^ University of ZadarZadarCroatia; ^9^ Institute of Marine Biology Kotor (IBMK)KotorMontenegro; ^10^ Department of Comparative Biomedicine and Food ScienceUniversity of PadovaLegnaroPadovaItaly

**Keywords:** 2b‐RAD, biophysical models, population genomics, sea urchin, seascape genetics, SNPs

## Abstract

Connectivity between populations influences both their dynamics and the genetic structuring of species. In this study, we explored connectivity patterns of a marine species with long‐distance dispersal, the edible common sea urchin *Paracentrotus lividus*, focusing mainly on the Adriatic–Ionian basins (Central Mediterranean). We applied a multidisciplinary approach integrating population genomics, based on 1,122 single nucleotide polymorphisms (SNPs) obtained from 2b‐RAD in 275 samples, with Lagrangian simulations performed with a biophysical model of larval dispersal. We detected genetic homogeneity among eight population samples collected in the focal Adriatic–Ionian area, whereas weak but significant differentiation was found with respect to two samples from the Western Mediterranean (France and Tunisia). This result was not affected by the few putative outlier loci identified in our dataset. Lagrangian simulations found a significant potential for larval exchange among the eight Adriatic–Ionian locations, supporting the hypothesis of connectivity of *P. lividus* populations in this area. A peculiar pattern emerged from the comparison of our results with those obtained from published *P. lividus* cytochrome b (cytb) sequences, the latter revealing genetic differentiation in the same geographic area despite a smaller sample size and a lower power to detect differences. The comparison with studies conducted using nuclear markers on other species with similar pelagic larval durations in the same Adriatic–Ionian locations indicates species‐specific differences in genetic connectivity patterns and warns against generalizing single‐species results to the entire community of rocky shore habitats.

## 1 Introduction

Population connectivity plays a key role in evolutionary and ecological processes that shape population dynamics and genetic structuring of species (Cowen, Paris, & Srinivasan, 2006; Puckett, Eggleston, Kerr, & Luettich, 2014). The ability to define the spatial scale at which population connectivity occurs is fundamental to enhancing our knowledge of dynamics and persistence of marine metapopulations and improving the success of biological resources management (Palumbi, 2003).

Most marine organisms (approximately 70% of marine invertebrates; Mileikovsky, 1971) display a bipartite life cycle characterized by a pelagic larval phase and sedentary adults. In these species, larval dispersal is expected to be the main process driving the exchange of individuals across seascapes and, consequently, ensuring population connectivity (D'Aloia et al., 2015; Gilg & Hilbish, 2003).

The small size of larvae, as well as the rapid dilution with distance and time from their natal origin, makes direct tracking of larval dispersal impractical (Cowen & Sponaugle, 2009). The study of genetic isolation patterns provides an indirect, often powerful, source of information for the inference of connectivity among populations (Hellberg, Burton, Neigel, & Palumbi, 2002; Palumbi, 2003; Thorrold et al., 2002). A review of 300 population genetic studies by Weersing and Toonen (2009) reported a weak correlation between average pelagic larval duration (PLD) and genetic differentiation (*F*_ST_) in marine taxa. In several cases, strong genetic partitioning was found despite a long PLD, indicating that marine populations can be less connected than expected. Limited connectivity between populations, even at small spatial scales (Taylor & Hellberg, 2003), may be explained by a number of mechanisms related to the biology of each species, such as larval behavior (Todd, 1998) and specific life‐history traits (Bowen, Bass, Muss, Carlin, & Robertson, 2006), or to ecological and environmental factors (Cowen & Sponaugle, 2009). Due to the complexity of the marine environment, Euclidean distance between locations is often a poor predictor of realized dispersal distance, because water circulation patterns and mesoscale features such as eddies and gyres can determine retention and/or diffusion of pelagic larvae at very different scales (Ruzzante, Taggart, & Cook, 1998; White et al., 2010).

Seascape genetics combines genetics and oceanography to investigate marine population connectivity by testing the role of environmental drivers in shaping the spatial genetic structure of species (Hansen & Hemmer‐Hansen, 2007; Storfer et al., 2006; White et al., 2010), thus allowing the comparison of the two approaches (Galindo, Olson, & Palumbi, 2006; Galindo et al., 2010; Selkoe et al., 2010).

**Figure 1:**
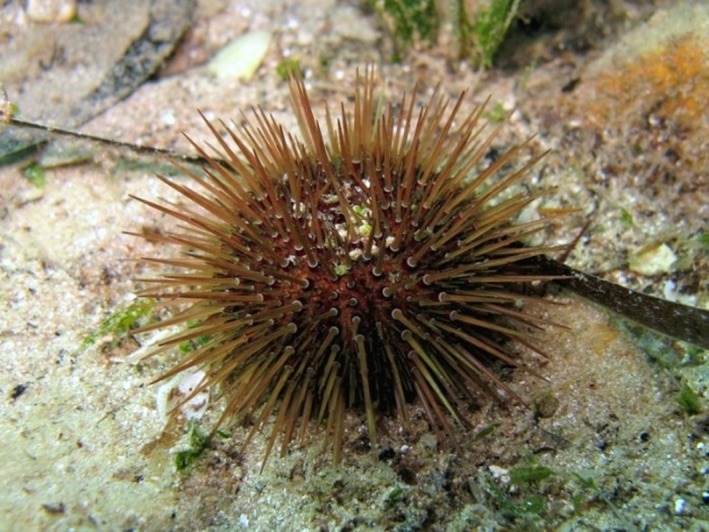
*Paracentrotus lividus* (source: Kroh & Mooi, 2017)

In our study, we applied a seascape genetics approach to explore connectivity patterns of a species with a long PLD, the edible common sea urchin *Paracentrotus lividus* (Lamarck, 1816; Figure 1), with special focus on populations in the Adriatic and Ionian seas. This geographic area was selected in the FP7 CoCoNET project (http://www.coconet-fp7.eu/) to investigate connectivity patterns of different species and to understand the scale at which marine protected areas (MPAs) can work as an effective network. We combined a population genomics approach with Lagrangian simulations produced by a biophysical model coupling oceanographic reanalyses (used as drivers for the movement of the planktonic stage) with information on life‐history traits affecting species dispersal. A set of genome‐wide single nucleotide polymorphisms (SNPs) was obtained using 2b‐RAD (Wang, Meyer, McKay, & Matz, 2012), a simple and cost‐effective restriction‐site‐associated DNA (RAD) sequencing method for genome reduction (Baird et al., 2008) aimed at the simultaneous identification and genotyping of thousands of SNPs evenly spread across the genome. This next‐generation sequencing approach provides a much higher resolution compared to those based on traditional markers, such as mitochondrial DNA sequencing or microsatellite genotyping. In addition, yielding a high number of loci scattered across the genome allows for the investigation into the presence of nonneutral processes of differentiation (Stapley et al., 2010) through the detection of outliers (Excoffier, Hofer, & Foll, 2009).

Echinoderms play a key role in structuring marine ecosystems. The sea urchin *P. lividus* is a keystone species in benthic communities of Mediterranean infralittoral rocky shores. Its grazing activity is considered one of the principal controlling factors of macroalgal community structure (Boudouresque & Verlaque, 2001; Privitera, Noli, Falugi, & Chiantore, 2011). This essential ecological role is threatened, because the species' gonads are considered a culinary delicacy, and therefore, sea urchins are heavily exploited at regional scales (Barnes & Crook, 2001). *P. lividus* is found in the sublittoral zone down to 20 m, throughout a distribution range that includes the Mediterranean Sea and the North‐East Atlantic Ocean from Ireland to the coasts of Morocco (Boudouresque & Verlaque, 2001). The larval stage (echinopluteus) is, on average, 30 days long (Fenaux, Cellario, & Etienne, 1985; Pedrotti, 1993).

The genetic structure of this species has been recently explored throughout its distribution range using mitochondrial and nuclear DNA sequencing (Calderón, Giribet, & Turon, 2008; Duran, Palacín, Becerro, Turon, & Giribet, 2004; Maltagliati, Di Giuseppe, Barbieri, Castelli, & Dini, 2010; Penant, Didier, Feral, & Chenuil, 2013). These studies detected the presence of two major genetic discontinuities: one between the Atlantic Ocean and the Mediterranean Sea, explained by the Almería‐Oran hydrological front (Calderón et al., 2008; Duran et al., 2004; Maltagliati et al., 2010), and the other one between the Adriatic Sea and the rest of the Mediterranean Sea (Maltagliati et al., 2010). By adding new mitochondrial and nuclear sequences and by reanalyzing published datasets, Penant et al. (2013) recently detected a signal of genetic differentiation at a smaller geographic scale, including differentiation within the Adriatic–Ionian seas.

In this context, thanks to their high frequency and genome‐wide coverage, SNP markers are expected to provide a more reliable and detailed picture of the genetic differentiation of *P. lividus*. Specifically, our study aims to: (1) evaluate the presence of large‐scale genetic structuring by comparing eight population samples from the Central Mediterranean (Adriatic and Ionian seas) with two population samples from the Western Mediterranean (i.e., France and Tunisia); (2) investigate the presence of genetic differentiation at a smaller scale by comparing samples within and between the Adriatic and Ionian basins; (3) estimate potential larval connectivity and retention via Lagrangian simulations based on a biophysical model developed for the Adriatic and Ionian basins; (4) compare the patterns obtained in (3) with the realized connectivity estimated via genetic analyses, in order to provide integrated and reliable data concerning the dispersal scale of *P. lividus*; (5) obtain useful information for the planning and management of MPAs across this geographic area by comparison with other target species.

## 2 Materials and Methods

### 2.1 Collection of samples, species confirmation, and total DNA extraction

**Figure 2:**
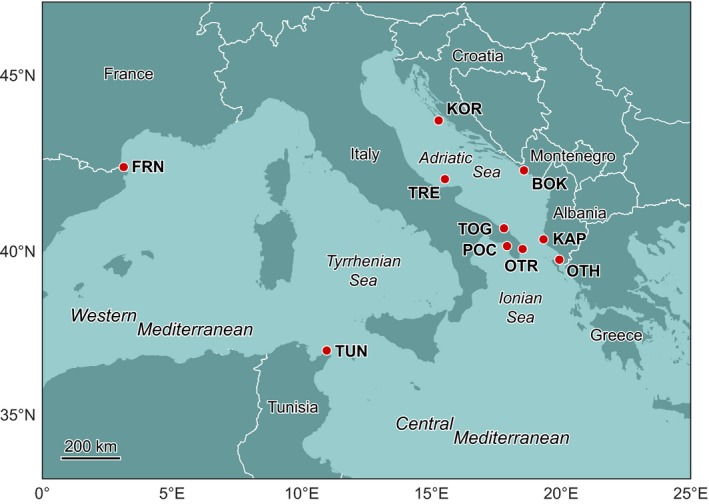
Sampling sites in the Central (Adriatic–Ionian seas) and the Western Mediterranean Sea (FAO subareas 37.1 and 37.2; FAO 2004). See Table 1 for location acronyms

A total of 275 individuals were collected at eight sampling locations in the Central Mediterranean (Adriatic–Ionian seas, FAO subarea 37.2; FAO 2004) and two locations in the Western Mediterranean (France and Tunisia, FAO subarea 37.1; FAO 2004) between April 2013 and October 2014 (Table 1 and Figure 2). Sampling locations in the Adriatic–Ionian seas were at nearest neighbors' distances between 70 and 300 km. The two additional population samples from France and Tunisia were included for comparison at a larger scale. At each location, sea urchins were collected by scuba divers. For each individual, 5–8 podia were removed and preserved in 95–96% ethanol until DNA extraction. Total genomic DNA (gDNA) was extracted using the Eurogold Tissue DNA Mini Kit (EuroClone), and its integrity was assessed by visualization on a 1% agarose gel stained with GelRed (BIOTIUM, GelRed™ Nucleic Acid Stain, 10,000× in Water). Concentrations and purity ratios of the samples (260/280 nm and 260/230 nm) were obtained by means of a NanoDrop UV–Vis spectrophotometer. When needed, samples were concentrated by precipitation with isopropanol (Sambrook, Fritsch, & Maniatis, 1990). Species identification was confirmed by PCR amplification and sequencing of about 1,000 bp (base pairs) of the cytochrome b gene (cytb) in at least 10 individuals from each population sample (*N* = 119). To this end, amplification and sequencing primers from Maltagliati et al. (2010) were used with the following PCR conditions: a total volume of 20 μl containing 2 mmol/L MgCl_2_, 0.2 μmol/L of each primer (Invitrogen), 250 μmol/L of each dNTPs, 1× Reaction Buffer (RBC Bioscience), 0.05 U/μl of *Taq* DNA polymerase (RBC Bioscience), 20 ng of DNA, and an amplification profile consisting of six touchdown cycles of 60 s at 94°C, 30 s at 62°C with a decrease of 0.7°C per cycle and 120 s at 72°C, followed by 24 cycles of 60 s at 94°C, 30 s at 58°C, and 120 s at 72°C, and by 5 min at 72°C for final extension. PCR products were purified using QIAquick PCR Purification Kit (QIAGEN) following the instructions of the supplier and sequenced by an external service (http://www.bmr-genomics.it/). Sequences were compared to NCBI database (blastn) to confirm identification of samples as *Paracentrotus lividus*.

**Table 1:** Sampling information of *Paracentrotus lividus* population samples examined in this study. For each population sample, sampling information about area, nation, sampling location, acronym, coordinates, date, and number of individuals *N* (processed/analyzed) are reported

Area	Nation	Sampling location	Acronym	Coordinates	Date	*N*
Ionian Sea	Greece	Othonoi Island	OTH	39.793289N 19.935636E	July 2013	31/31
Ionian Sea	Albania	Karaburun Peninsula	KAP	40.392800N 19.324967E	June 2013	30/30
Adriatic Sea	Montenegro	Boka Kotorska	BOK	42.387533N 18.569633E	June 2013	26/25
Adriatic Sea	Croatia	Kornati Islands	KOR	43.792250N 15.281483E	June 2013	26/26
Adriatic Sea	Italy	Tremiti Islands	TRE	42.138583N 15.523950E	April 2013	29/28
Adriatic Sea	Italy	Torre Guaceto	TOG	40.716650N 17.800050E	May 2013	31/31
Adriatic–Ionian Sea	Italy	Otranto	OTR	40.109233N 18.519217E	May 2013	30/30
Ionian Sea	Italy	Porto Cesareo	POC	40.195250N 17.917950E	May 2013	30/29
Western Med. Sea	France	Banyuls	FRN	42.482290N 3.1374160E	October 2014	26/26
Western Med. Sea	Tunisia	Haouaria	TUN	37.050440N 10.967000E	January 2014	16/16

### 2.2 Construction of 2b‐RAD libraries

The 2b‐RAD technique (Wang et al., 2012) was tested on six samples and optimized for processing *P. lividus* gDNA (2b‐RAD oligonucleotide sequences used for Illumina sequencing are reported in Table S1). Following this preliminary test, about 400 ng of high‐quality RNA‐free gDNA from each individual (*N* = 275) was cleaved with 2 U of the type 2b restriction endonucleases *Csp*CI (New England BioLabs) overnight at 37°C, producing a population of fragments of uniform length (35 bp) with protruding ends (Marshall & Halford, 2010). For each sample, 1 μl of digested DNA was loaded on a 1% agarose gel stained with GelRed™ alongside a comparable amount of intact genomic DNA from the same sample to check the performance of the restriction reaction. The digested products were ligated to partially double‐stranded adaptors with compatible and fully degenerated overhangs in a 25‐μl total volume reaction consisting of 0.4 μmol/L of each adaptor, 0.2 mmol/L ATP, and 40 U/μl T4 DNA ligase (SibEnzyme). The 2b‐RAD tags were amplified for a few cycles (16) using two pairs of primers to amplify and to introduce sample‐specific barcodes (7 bp) and the annealing sites for Illumina next‐generation sequencing. Barcodes were designed by Barcode Generator (available at UC Davis Web site: http://comailab.genomecenter.ucdavis.edu/index.php/Barcode_generator). Amplification PCR consisted of 10 μl of ligated DNA, 0.2 μmol/L of each primer 2b‐RAD amp, 0.5 μmol/L of each primer F and barcoded primer R, 0.3 mmol/L dNTPs, 1× Phusion HF buffer, and 0.02 U/μl Taq Phusion high‐fidelity polymerase (New England BioLabs) in a total volume of 50 μl. Amplification products were run on 1.8% agarose gel stained with GelRed™ to check the quality of reactions. PCR products (1–10 μl) of different individuals were pooled according to the concentration of the target band (about 170 bp, including the restriction fragment of interest and the adaptors/barcodes). The amount of the PCR product from each individual was estimated by combining the concentration values obtained from NanoDrop UV–Vis spectrophotometer and the target band intensity obtained from agarose gel using ImageJ (Schneider, Rasband, & Eliceiri, 2012). A total of three pools (about 90 barcoded samples per pool) were assembled. The target band of about 170 bp was purified by removing contaminating fragments (high molecular weight fragments and primer–dimers) in two consecutive steps. First, each pool was run on 1% agarose gel, and the target band was excised and eluted in distilled water overnight; second, a further purification of each pool was performed using magnetic beads (SPRIselect, BECKMAN COULTER) according to the solid‐phase reversible immobilization (SPRI) method (DeAngelis, Wang, & Hawkins, 1995).

### 2.3 2b‐RAD tag sequencing

Each pool was sequenced on Illumina HiSeq platforms with a single‐end SR50 High Output mode by UC Davis Genome Center (CA, USA) or Genomix4Life S.r.l. (Baronissi, SA, Italy), which also performed data demultiplexing and quality filtering. Each pool was sequenced twice: following sequencing results of the first run, the relative amount of the target 170 bp band of each individual was precisely estimated based on the number of reads obtained for each sample. A new pool was then assembled for each group of individuals, adjusting volumes to obtain the final normalization. The new pool was purified and sequenced as before, and the reads of each individual from the first and second Illumina sequencing run were merged and analyzed together. This procedure allowed for an increase in the depth coverage and ensured obtaining an equal and comparable number of reads from each analyzed sample.

### 2.4 *De novo* analysis: in silico identification of loci and genotyping

The quality of raw demultiplexed reads was checked with FastQC software (available at http://www.bioinformatics.babraham.ac.uk/projects/fastqc/); then, custom‐made scripts were used to filter reads for the presence of *Csp*CI recognition sites and to trim adaptors, obtaining sequences of uniform length (32 bp). The trimmed, high‐quality reads (mean quality score per base > 37) were used for subsequent analysis. In silico assembly of loci and genotyping was performed using STACKS software (Catchen, Hohenlohe, Bassham, Amores, & Cresko, 2013) employing the “denovo_map.pl” pipeline. A technical replicate of one sample was used to estimate error rates and to optimize *de novo* assembly parameters of 2b‐RAD data. The parameters were set to: minimum stack depth (*m*) of 15; number of mismatches allowed between stacks to build a locus in an individual (*M*) equal to 4; maximum distance between loci from distinct individuals to be merged in the population catalog (*n*) equal to 4; error rate to call SNP (*bound*) between 0 and 0.1; significance level required to call a heterozygote or homozygote (*alpha*) equal to 0.05. The STACKS module *Populations* was used to generate output in GENEPOP format for further downstream analysis. As the minimum coverage for each allele per locus was set to 15 (parameter *m*, see above), no further filtering by sequencing depth was applied. CREATE software (Coombs, Letcher, & Nislow, 2008) was used for conversion of the GENEPOP input file into input formats for different genetic analysis programs. The final dataset included all polymorphic loci present in at least 80% of the individuals, and it was characterized by the presence of 1–3 SNPs and 2–6 alleles. When multiple SNPs were found at one locus, only the SNP with the highest expected heterozygosity (proxy for polymorphic content, Phillips, 2005) across the whole dataset was retained. The threshold of missing loci per individual was set to 30%.

### 2.5 SNPs validation by Sanger sequencing

To evaluate the accuracy of the 2b‐RAD protocol and to verify the existence and polymorphism level of candidate SNPs, a preliminary STACKS run of 136 individuals was used to select a reduced number of loci for validation by a PCR‐based approach and Sanger sequencing. An available dataset of more than 140,000 EST‐linked sequences of *P. lividus* was downloaded from the NCBI database (four libraries; accession numbers: MPMGp1171–1174) and used to create a local database. Consensus sequences of a subset of 2b‐RAD loci with different polymorphism levels were used as query for a local blastn against the EST‐linked sequences database. Consensus sequences of each locus and its matching EST‐linked sequences were aligned using CLUSTALW Omega tool (available online at http://www.ebi.ac.uk). Primers for Sanger sequencing were designed on a selection of 10 loci using sequence information of alignments and tested on a small number of individuals with a known genotype. PCR conditions were optimized depending on annealing temperature of each pair of primers and on the expected amplicon length (primer sequences used for validated loci are reported in Table S2).

### 2.6 Analysis of population genomics data

The genetic variability within population samples was assessed on polymorphic loci by computing the observed heterozygosity (*H*_o_) and unbiased expected heterozygosity (*H*_e_) with GENETIX 4.05.2 (Belkhir, Borsa, Chikhi, Raufaste, & Bonhomme, 2000). Allelic richness (*A*_R_) was calculated using HP‐RARE (Kalinowski, 2005) based on the smallest sample size (*N* = 16) across all population samples.

The analyses of population differentiation and population genetic structuring were performed using different programs. ARLEQUIN 3.5 (Excoffier & Lischer, 2010) was used to calculate pairwise genetic distances between populations (*F*_ST_), as well as nonhierarchical and hierarchical analysis of molecular variance (AMOVA; Excoffier, Smouse, & Quattro, 1992); in the latter case, all the possible partitions of populations in two and three groups were tested. Considering the presence of missing data, due to our filtering strategy, all the analyses were performed using the locus‐by‐locus option in ARLEQUIN. Significance levels for multiple comparisons were adjusted according to Benjamini and Hochberg (1995) correction for multiple tests. The package ADEGENET for R 3.2.3. (Jombart, 2008) was used for discriminant analysis of principal components (DAPC), a multivariate method to represent clusters of genetically related individuals providing a useful visual assessment of between‐population differentiation.

Given the genetic homogeneity found in the Adriatic–Ionian basins (see Section 3), we carried out forward‐time simulations with SimuPOP (Peng & Kimmal, 2005) using different mutation rates and population sizes, to determine the number of generations and the amount of gene flow needed to achieve the *F*_ST_ observed in the empirical dataset. We modeled eight ideal populations using the overall SNP frequencies of the Adriatic–Ionian samples. A universal standard mutation rate (2.5 × 10^−8^ mutation per generation; Pontes et al. 2015) and a faster mutation rate (1 × 10^−6^) were tested. A wide range of population sizes was used (100–10,000), including the smallest effective population size (*N*_e_) value estimated from *P. lividus* microsatellites temporal variation (100 individuals; Calderón, Palacín, & Turon, 2009), new estimates obtained from our SNPs with the linkage disequilibrium method by NeEstimator (Do et al., 2014; Table S4) and arbitrarily high values. Two alternative scenarios of isolation and migration were tested. First, the simulations were run under a pure drift model (0 migrants per generation), and the number of generations of divergence needed to reach and exceed the observed *F*_ST_ was recorded. Second, the simulations were performed under a scenario of migration; in this case, the number of generations and the number of migrants per generation needed to reach an equilibrium *F*_ST_ value close to the observed one were recorded. The complete set of parameters for the different scenarios is reported in Table S5.

### 2.7 Outlier detection

Population genetics analyses typically assume that the markers employed are selectively neutral. To detect loci under directional selection, we used the *F*_ST_‐outlier method implemented in LOSITAN (Antao, Lopes, Lopes, Beja‐Pereira, & Luikart, 2008) and the Bayesian approach of BAYESCAN 2.01 (Foll & Gaggiotti, 2008). LOSITAN was run with the following settings: 1 million simulations under neutral mean *F*_ST_, confidence interval of 0.95%, a false discovery rate (FDR) of 0.01, and the infinite allele model; for each run, three replicates were performed. BAYESCAN was run with burn in = 50,000, thinning interval = 30, sample size = 5,000, number of pilot runs = 50, length of pilot runs = 5,000, and the same false discovery rate (FDR) threshold set in LOSITAN (0.01). In both cases, all the polymorphic loci were used and the comparisons were performed between Adriatic–Ionian population samples and the two additional population samples from the Western Mediterranean Sea, as well as among Adriatic–Ionian population samples. The allele sequences of all loci identified as putative outliers were searched against a *P. lividus* transcriptome database of 188,000 contigs (NCBI database, accession number GCZS00000000.1; Gildor, Malik, Sher, Avraham, & Ben‐Tabou de‐Leon, 2016) using a local blastn with an e‐value cutoff of 3 × 10^−6^. The matching contigs of *P. lividus* transcriptome were compared to the NCBI nucleotide collection, and the top blastn hits were selected.

### 2.8 Lagrangian simulations of larval dispersal in the Adriatic and Ionian basins

Potential connectivity of *P. lividus* among the eight Adriatic and Ionian locations at which genetic samples were collected was assessed by Lagrangian simulations, using a biophysical model developed to investigate larval dispersal in the Adriatic–Ionian basins (Melià et al., 2016; Schiavina, Marino, Zane, & Melià, 2014). As a thorough investigation of connectivity across the whole Adriatic–Ionian basin would require basin‐wide habitat mapping, which is not available at present for *P. lividus*, we adopted a conservative approach and assessed only direct connections among the limited number of locations used for genetics. The existence of additional unmodeled *P. lividus* populations, due to the availability of suitable rocky shore habitats across the surveyed area, is expected to further increase the potential for dispersal among the modeled locations through stepwise processes, making our estimates of connectivity robust (and estimates of isolation questionable).

The oceanographic engine of the biophysical model is based on the ocean circulation dataset produced by the AREG model (Guarnieri, Oddo, Pastore, Pinardi, & Ravaioli, 2010; Oddo, Pinardi, Zavatarelli, & Coluccelli, 2006) and provided by the Adriatic Forecasting System (http://oceanlab.cmcc.it/afs). Daily average fields of current velocity and temperature cover the whole Adriatic basin down to the 39°N parallel in the Ionian Sea over a regular horizontal grid with a resolution of 1/45° (about 2.2 km) and 31 vertical sigma layers. Bathymetry has a horizontal resolution of 1/60°, and the coastline is set in correspondence with the 10‐m isobath.

The biological component of the model was based on the available knowledge about key life‐history traits affecting larval dispersal. Considering an age at capture for sampled individuals that varies between 0 (i.e., newly recruited) and 9 years (i.e., maximum age; Crapp & Willis, 1975), our simulations spanned the period when they were likely born, that is 2004–2013. Spawning was assumed to occur daily between April and July (Sellem & Guillou, 2007), except in days when water temperature exceeded 18°C (Spirlet, Grosjean, & Jangoux, 1998). One thousand Lagrangian particles (representing individual larvae) were released each spawning day from each sampling location. Released particles were uniformly distributed along a depth from 0.5 to 10 m (Boudouresque & Verlaque, 2001) at random positions distributed according to a 2‐D Gaussian spatial distribution centered at each location with a standard deviation of 1 km. Pelagic larval duration was assigned at birth to each individual and drawn from a Gaussian distribution with mean 30 (±5) days (Fenaux et al., 1985; Pedrotti, 1993). Trajectories were stepped forward at fixed depth via an explicit fourth‐order Runge–Kutta integration method with a very fine temporal step (6 min) and followed until the end of the pelagic phase or larval death. Larval mortality was considered to be triggered by water temperature: above 18°C, we introduced a mortality rate μ_L_ = 3 d^−1^, corresponding to a survival probability σ_L_ = 5% per day (Privitera et al., 2011). Connectivity effectiveness (*sensu* Melià et al., 2016) was calculated as the ratio (averaged over the simulation period) between the number of particles successfully moving from one location to another and the number of particles released from the location of origin. Larvae were considered to successfully colonize the destination location if they survived their pelagic phase and their final position fell within a circular buffer with a 5‐km radius from the destination location. Connectivity persistence (measuring the continuity of the flux throughout the years, *sensu* Melià et al., 2016) was defined as the stabilization coefficient (that is the reciprocal of the coefficient of variation) of the flux calculated over the simulation period. Time series of release, survival, and success rates were tested for possible trends over the 10 years of simulation via a modified Mann–Kendall test (Hamed & Rao, 1998).

### 2.9 Comparison with published cytb data

As mentioned in Introduction, Penant et al. (2013) provided evidence for significant differentiation on a small geographic scale. For Adriatic and Ionian samples, differentiation was found by reanalyzing published mitochondrial cytb sequences (Maltagliati et al., 2010). Differences were stronger using frequency‐based *F*_ST_ than using φ_ST_, possibly reflecting strong drift effects at this marker that would produce significant *F*_ST_ in few generations (Penant et al., 2013).

Considering that no differences were found for Adriatic and Ionian samples in our study using SNPs (see Section 3), and the opposite was found in Penant et al. (2013) we evaluated the statistical power to detect predefined levels of genetic differentiation in the two studies. To this end, we used the forward‐time simulation method of Ryman and Palm (2006), implemented in the software POWSIM 4.1, which allows simulation of sampling from populations at various levels of expected divergence using a classical Wright–Fisher model of drift without migration or mutation. We performed two sets of simulations with POWSIM, one using the cytb haplotype frequencies and sample sizes of the seven Adriatic–Ionian populations (*N* = 10 each) reported in Penant et al. (2013), and the other based on the SNPs frequencies and sample sizes of our eight Adriatic–Ionian population samples. We used a range of population sizes to estimate the number of generations needed, in the case of pure genetic drift, to produce the global *F*_ST_ value observed with the cytb sequences and the power to detect differences with this marker. We compared these results with those obtained with our SNPs panel using the same number of generations but a fourfold greater effective population size compared with that used for mtDNA, as expected in the case of a balanced sex ratio for diploid markers (Hedrick, 2011). For each run, 200 replicates were performed. In the case of SNPs, the executable Powsim_b was used to accommodate more than 1,000 loci.

## 3 Results

### 3.1 DNA extraction and species identification

The extraction protocol allowed successful extraction of intact, RNA‐free genomic DNA from all 275 collected samples. A total of 119 partial cytb coding sequences of about 1,000 bp were obtained by Sanger sequencing; the blastn search against the NCBI nucleotide database confirmed the identification as *Paracentrotus lividus* of all tested specimens.

### 3.2 Sequencing results, filtering, and selection of loci for genetic analyses

A total of 1,076,343,894 demultiplexed and filtered quality reads were obtained from Illumina sequencing of the three 2b‐RAD pools (*N* = 276; one technical replicate was included). After trimming and filtering for restriction site, a total of 992,809,025 reads of 32‐bp length were retained (92%), resulting in about 3.5 million reads per individual. An average of 25,000 tags per individual was obtained, leading to an approximate coverage per locus of 140. In general, a uniform read coverage between samples for the same locus was observed, whereas variability was observed between different loci. A total of 212,260 2b‐RAD tags were identified among 276 individuals, 138,745 (65%) were monomorphic and 73,515 (35%) were polymorphic. After filtering, we retained 1,122 polymorphic loci for further analysis. The technical replicates matched at 1,112 of 1,122 loci; nine of ten differences were due to missing loci between the two replicates. Of the 1,122 loci, 261 loci had only one SNP, 420 had two SNPs, and 441 had three SNPs, for a total of 2,424 SNPs. As mentioned in Section 2, when a locus had 2 or 3 SNPs, only the SNP globally showing the highest expected heterozygosity was retained. The replicated individual and three individuals showing more than 30% of missing loci were excluded a posteriori from the analysis. The remaining 272 individuals shared on average 95% of loci. Lastly, the consensus sequences of these 1,122 loci were employed in a local blastn against the mitochondrial genome of *P. lividus* (NCBI accession NC_001572; Cantatore, Roberti, Rainaldi, Gadaleta, & Saccone, 1989). As no matches were found with the mitochondrial sequences, the 1,122 loci can be considered as nuclear markers.

### 3.3 SNPs validation by Sanger sequencing

The consensus sequences of 179 loci from a preliminary run were used for a local blastn search against the EST‐linked sequences dataset, and a total of 28 matches were identified. The alignment of each locus and its matching EST‐linked sequences were analyzed, and 10 loci with different polymorphism level were chosen for the primers design. PCR conditions were optimized for each locus, and nine loci were successfully amplified on a panel of 4–6 individuals. The PCR products of five loci were too long to be sequenced (>2,000 bp); considering that PCR primers were designed on EST‐linked sequences, this result probably reflects the presence of introns within these PCR products. The remaining four loci were successfully sequenced, and the 2b‐RAD genotypes were confirmed (Table S2).

### 3.4 Genetic analyses

Comparable values were obtained among all the population samples for observed heterozygosity, unbiased expected heterozygosity, and allelic richness (Table S3), with one‐way ANOVA showing no significant differences among samples (*p* > .05).

#### 3.4.1 Population structure

**Figure 3:**
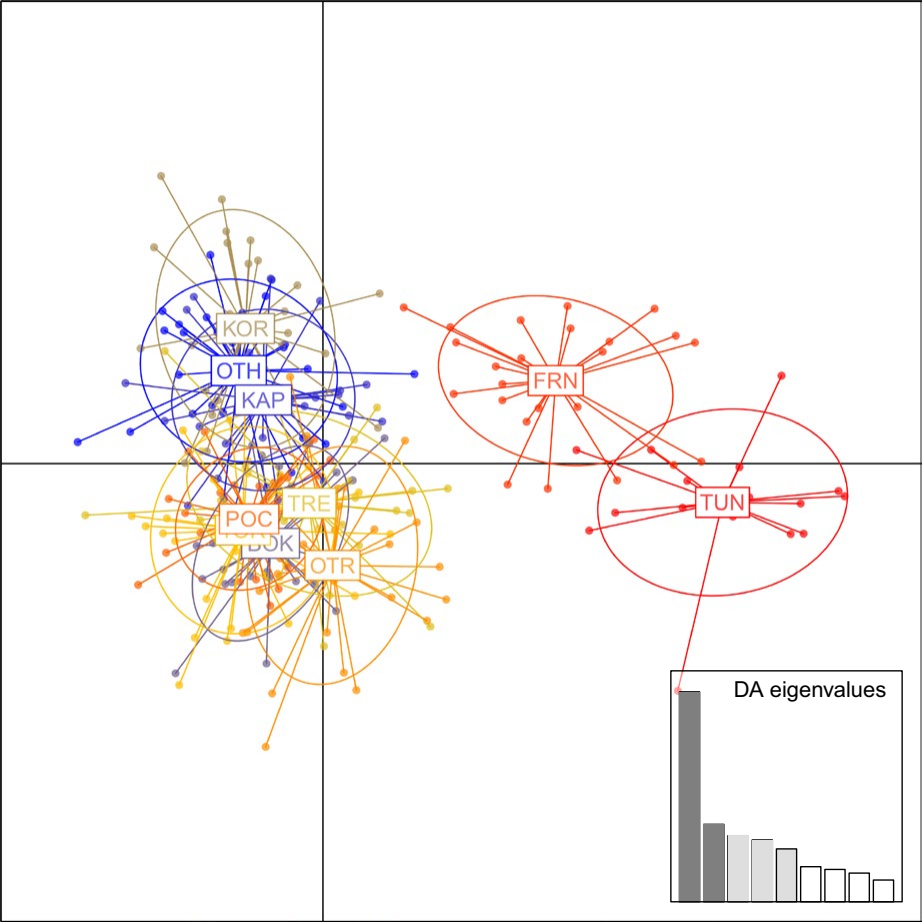
Discriminant Analysis of Principal Components (DAPC) performed by package ADEGENET. See Table 1 for location acronyms

Considering all ten population samples from the Central Mediterranean (Adriatic and Ionian seas) and Western Mediterranean (France and Tunisia), the analysis of molecular variance showed that most of the variation (>99%) occurred within populations and only 0.63% among populations. However, the corresponding global *F*_ST_ was highly significant (*p* < .0001), and its 95% confidence interval (CI) did not include 0 (0.00435–0.00828), indicating the presence of genetic structuring across the study area. All sixteen pairwise *F*_ST_ comparisons between Adriatic–Ionian and the Western Mediterranean population samples were highly significant, with *F*_ST_ values ranging from 0.00663 to 0.02475 (.0001 < *p* < .0023; significant after Benjamini and Hochberg correction for multiple tests; Table 2) and 95% CIs ranged from 0.00211 to 0.03357. In the Western Mediterranean, the comparison between France and Tunisia (*F*_ST_ = 0.01013, *p* = .0021) was also highly significant after correction for multitest. Hierarchical AMOVA showed that the genetic variation can be partitioned into three geographic groups, the first including the eight Adriatic–Ionian population samples, the second including only the population sample from France, and the third one including the sample from Tunisia (*F*_CT_ = 0.01334; *p* < .0001); also in this case, the 95% CI did not include 0 (0.0089–0.01823). This subdivision into three different geographic pools maximized the amount of variation among groups with respect to any alternative configuration including either two or three groups. Notably, the subdivision into two groups as proposed in the literature (Central vs. Western Mediterranean, Maltagliati et al., 2010) was still significant, although with a smaller *F*_CT_ (*F*_CT_ = 0.01101; *p* < .0001). This genetic pattern was confirmed by DAPC (Figure 3), which shows that samples pooled into three groups: one comprising all the samples from the Central Mediterranean, one associated with the population sample from France, and one including the population sample from Tunisia.

**Table 2:** Pairwise genetic distances (*F*_ST_) between Adriatic–Ionian samples and samples from France and Tunisia based on 1,122 polymorphic loci. Benjamini & Hochberg correction for multiple tests was applied. *F*_ST_ indices and *p*‐values are reported below and above the diagonal, respectively; significant indices in bold. Comparisons between Adriatic–Ionian populations in gray. See Table 1 for location acronyms

	OTH	KAP	BOK	KOR	TRE	TOG	OTR	POC	FRN	TUN
**OTH**		.2658	.3513	.7535	.2974	.0343	.0562	.4461	**<.0001**	**<.0001**
**KAP**	.00244		.0707	.9170	.5003	.8506	.4594	.3562	**.0023**	**<.0001**
**BOK**	.00283	.00508		.1430	.3254	.5018	.3289	.0690	**<.0001**	**<.0001**
**KOR**	.00053	−.00064	.00507		.8482	.5956	.8599	.6084	**<.0001**	**<.0001**
**TRE**	.00257	.00166	.00329	.00015		.2869	.4514	.9383	**<.0001**	**<.0001**
**TOG**	.00495	.00003	.00245	.00170	.00288		.8191	.8147	**<.0001**	**<.0001**
**OTR**	.00472	.00203	.00353	.00038	.00233	.00061		.6421	**<.0001**	**<.0001**
**POC**	.00180	.00227	.00559	.00151	−.00075	.00050	.00147		**.0001**	**<.0001**
**FRN**	**.01264**	**.00663**	**.01633**	**.01122**	**.01121**	**.00984**	**.01488**	**.01093**		**.0020**
**TUN**	**.02475**	**.01999**	**.01886**	**.02029**	**.01612**	**.02350**	**.01882**	**.02233**	**.01013**	

At the basin scale, by contrast, pairwise *F*_ST_ values among the eight Adriatic–Ionian population samples were all negligible (*F*_ST_ ranging from −0.00075 to 0.00559) and statistically not significant after multiple test correction (Table 2, gray cells). The AMOVA conducted on the Adriatic–Ionian population samples confirmed the pattern of genetic homogeneity at the basin scale (*F*_ST_ = 0.00217, *p* = .4171).

SimuPOP forward‐time simulations revealed that, in the case of complete isolation under pure drift, the observed *F*_ST_ of Adriatic–Ionian population samples can be reached and exceeded in a few generations (Table S5). In particular, a maximum of 45 generations was necessary to reach and exceed the Adriatic–Ionian *F*_ST_ (0.00217). This maximum value was obtained using a population size of 10,000, whereas considering a value of 100 (*N*_e_ from microsatellite temporal variation; Calderón, Palacín, et al., 2009) and 1,000 (the biggest *N*_e_ calculated from our data; Table S4), the observed *F*_ST_ was obtained in as few as 1–5 generations. On the other hand, in a scenario of migration, 5–15 migrants were sufficient to achieve and consistently maintain the observed *F*_ST_. In this case, the equilibrium was reached in as few as 2–15 generations when the population size was set to 100–1,000 individuals, or in a maximum of 120 generations for extreme, perhaps unrealistic, population sizes (Table S5). All these results were not affected by the use of different mutation rates.

#### 3.4.2 Outlier detection

To investigate whether the differentiation signal was generated by one or few loci putatively under positive selection, the dataset of 1,122 polymorphic loci was explored both with LOSITAN and BAYESCAN. After FDR correction and considering the results obtained in all three replicates, LOSITAN identified a total of 17 loci as putative outliers under positive selection (high *F*_ST_/*H*_E_ ratio) when comparing Central and Western Mediterranean population samples (Table S6). Specifically, five outliers were identified between Adriatic–Ionian population samples and France, and 12 between Adriatic–Ionian samples and Tunisia. No outliers were detected when comparing Adriatic–Ionian population samples. Conversely, BAYESCAN did not identify any locus as a putative outlier under positive selection. Interestingly, nine of the 17 putative outliers identified by LOSITAN had a match with the transcriptome of *P. lividus*, showing 93%–100% of query identity (30–32 bp). Due to some redundancy in *P. lividus* transcriptome, in six of the nine cases (asterisk in Table S6), the allelic sequences of putative outliers displayed a match with more than one contig, but these were identical and even so all alleles of each outlier pointed to the same contig hits. Six of these nine transcripts displayed a top blastn hit with *Strongylocentrotus purpuratus* proteins in the NCBI nucleotide database, and notably one of these transcripts revealed 99% query coverage and 76% identity with the codifying sequence of the hyalin gene (Table S6).

The pattern of genetic structure obtained with all 1,122 loci was confirmed also when excluding the 17 putatively selected outliers from the dataset (Table S7). For instance, compared to the dataset comprising all 1,122 loci, a slight reduction in the variation among the 10 populations was found with the 1,105 putative neutral loci (global *F*_ST_ = 0.00551, *p* < .0001). Similarly, the hierarchical AMOVA on the three groups identified above (Adriatic–Ionian samples, France, and Tunisia) confirmed the genetic structure obtained with the full dataset (*F*_CT_ = 0.01067, *p* < .0001). The pairwise *F*_ST_ between Central and Western Mediterranean samples and between France and Tunisia remained small but highly significant after correction for multitest, and comparisons among Adriatic–Ionian samples were all nonsignificant.

### 3.5 Lagrangian simulations of larval dispersal in the Adriatic and Ionian basins

**Figure 4:**
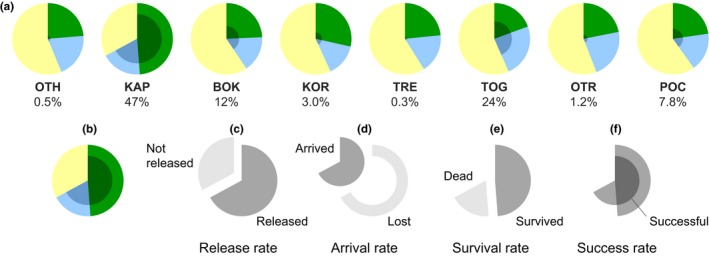
Geographic variation of connectivity among sampling locations as obtained from the biophysical model. The pie charts in the first row (a) detail the factors determining the success rate associated with each sampling location (see Table 1 for location acronyms), expressed as the percentage of successful larvae over the actual release, averaged over the whole simulation period. Pies in the second row (b–f) serve as a legend, using the KAP pie (b) as an example. The actual fraction of larvae released from a sampling location (c, release rate) depends on the presence of favorable thermal conditions for spawning (see text). Only a fraction of the larvae that are actually released reach other locations (d, arrival rate) or survive the dispersal phase (e, survival rate). The fraction of successful larvae dispersing from the location of release to any other location (f, success rate) is eventually obtained as the intersection between larvae arrived (dark gray slice in d) and larvae survived (dark gray slice in e)

The strength of each sampling location to serve as a direct source of propagules for other locations is summarized through pie diagrams in Figure 4. The proportion of actual released larvae (Figure 4) corresponds to the fraction of the reproductive period in which water temperature in the source location was favorable to spawning (see Section 2). This fraction ranged between 40% (POC) and 67% (KAP), with an average across locations equal to 46%. Survival rate (i.e., the fraction of larvae that were not killed by mortality events triggered by water temperatures >18°C) reflected thermal variation experienced by larvae during their dispersal and ranged between 20% (TOG) and 49% (KAP) of the actual release from each location, with an average across locations of 27%. Success rate (i.e., the fraction of released larvae which survived until reaching a destination location at the end of their PLD) was on average 7% of the actual release and ranged, with a conspicuous variation spanning orders of magnitude from location to location, between 0.3% (TRE) and 47% (KAP).

Estimates of connectivity effectiveness (Table 3) and persistence (Table 4) for successful particles, as derived from Lagrangian simulations, are reported in matrices whose rows/columns refer to the eight sampling locations of the Adriatic–Ionian seas (see again Figure 2) ordered in a counterclockwise direction from OTH to OTR. Retention (i.e., self‐connectivity, represented by the diagonal elements of the matrix) was lowest (<1%) in three locations (OTH, TRE, OTR), intermediate (between 1% and 20%) in two (BOK, POC), while it was highest (>20%) in TOG and KAP. Self‐connections had also higher levels of persistence compared to cross‐connections, with the exception of TRE. All sampling locations were connected to the nearest location in the counterclockwise direction, as revealed by the first elements above the diagonal in the matrix of connectivity effectiveness (Table 3). Those elements were systematically larger than the corresponding ones below the diagonal (which represent connections in the opposite direction); the only exception was the connection between TRE and KOR, in which the exchange of particles from west to east was stronger (in terms of both effectiveness and persistence) than that from east to west. It is apparent that only a few off‐diagonal elements of the matrices in Tables 3 and 4 are not null. In terms of larval connectivity for *P. lividus* in the focal area, this means that only in some cases do the propagule fluxes directly connect sampling locations that are not nearest neighbors. Particles originating from Greek coasts (OTH) were able to reach Montenegro (BOK), and particles from both OTH and KAP were successful in crossing the Adriatic Sea and reaching some Italian sampling locations (OTR and POC). All Italian locations were connected with each other from north to south (TRE to POC), in most cases through persistent (>0.75) connections. TRE acted as an important source of particles for all Italian locations, while POC acted as an effective receptor of particles coming from most locations on both sides of the basin (with the exception of the two northernmost locations on the eastern side, BOK and KOR). Some particles were occasionally able to cross the Adriatic Sea from west to east, starting from TOG and OTR.

**Table 3:** Connectivity effectiveness for *Paracentrotus lividus*. Connectivity effectiveness (estimated via Lagrangian simulation) is measured as the proportion (averaged over the simulation period) of larvae successfully moving from the locations of origin (in rows) to the destination locations (in columns) with respect to the potential releases. Positive values are reported as percents. Shaded cells indicate retention (i.e., self‐connectivity). See Table 1 for location acronyms

	OTH	KAP	BOK	KOR	TRE	TOG	OTR	POC
**OTH**	0.224	0.269	0.002	–	–	–	0.005	0.015
**KAP**	0.029	47.077	0.036	–	–	–	0.007	0.001
**BOK**	–	0.001	12.446	0.003	–	–	–	–
**KOR**	–	0.000	–	2.997	0.006	–	–	–
**TRE**	–	0.000	–	0.019	0.002	0.197	0.054	0.043
**TOG**	–	0.002	0.003	–	–	23.869	0.046	0.030
**OTR**	0.001	0.063	0.001	–	–	0.003	0.660	0.474
**POC**	–	–	–	–	–	–	–	7.759

**Table 4:** Connectivity persistence for *Paracentrotus lividus*. Connectivity persistence is defined as the stabilization coefficient (i.e., the reciprocal of the coefficient of variation) of the average annual flux of larvae successfully moving from the locations of origin (in rows) to the destination locations (in columns), calculated over the simulation period. Shaded cells indicate retention. See Table 1 for location acronyms

	OTH	KAP	BOK	KOR	TRE	TOG	OTR	POC
**OTH**	1.326	0.651	0.444	–	–	–	0.441	0.649
**KAP**	0.671	2.293	0.357	–	–	–	0.667	0.433
**BOK**	–	0.316	3.962	0.463	–	–	–	–
**KOR**	–	–	–	1.284	0.316	–	–	–
**TRE**	–	–	–	0.809	0.316	1.236	1.177	0.384
**TOG**	–	0.454	0.316	–	–	1.914	0.962	0.834
**OTR**	0.316	0.465	0.316	–	–	0.394	1.365	0.824
**POC**	–	–	–	–	–	–	–	2.039

**Figure 5:**
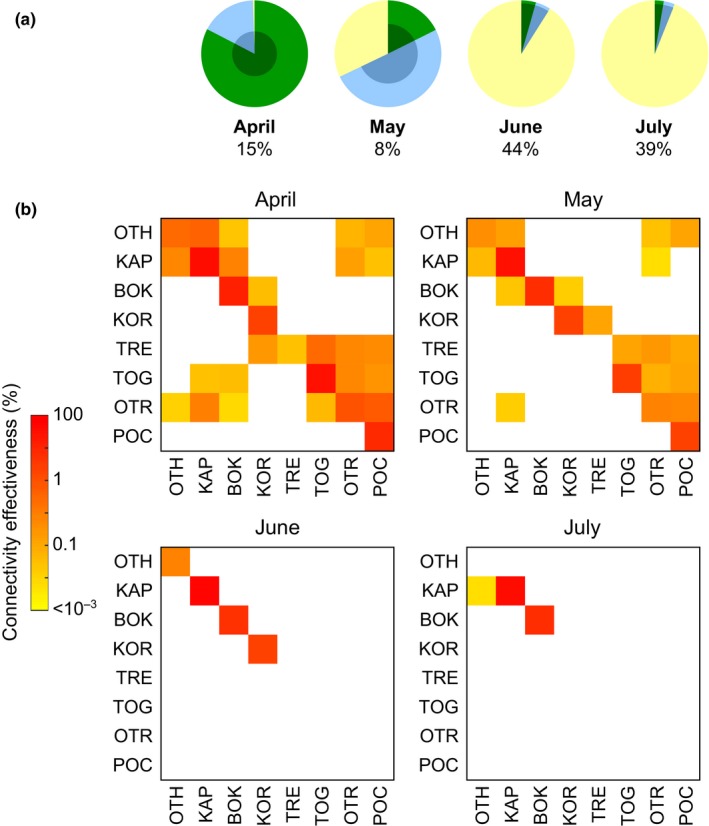
Monthly variation in potential connectivity as obtained from the biophysical model. (a) Success rate over the actual release (averaged over years and sampling locations; see Figure 4 for color codes). (b) Connectivity effectiveness matrices (averaged over years; see Table 1 for location acronyms)

Changes in connectivity throughout the reproductive season following the effect of changing water temperature are shown in Figure 5. Release rate (averaged across all the release locations) decreased from April to July (approximately from 99% to 6%, Figure 5a). It was quite similar in all locations but KAP, where lower water temperatures determined release rates as high as 40% also in June and July. Survival rate (which is affected by the temperature experienced by particles along their trajectories) followed a nonlinear pattern over the reproductive season, with a maximum in April (83%) and a minimum in May (26%). Success rate, as a result of the interaction between seasonal variation in water temperature and current patterns, varied between a minimum of about 8% in May and a maximum in June and July (44%) due to high rates of particle arrival in summer months. The effect of temperature on the connectivity effectiveness matrix is shown in Figure 5b. Connectivity patterns were richer in April and May, while in June and July they were reduced to a few self‐connections on the eastern side of the basin. The analysis of interannual variation of release, survival, and success rates showed no significant trend in release and success rate (*p* > .2), while survival showed a significant (*p* = .01) negative trend (*y* = −0.015*t* + 31.05), suggesting that changes in water temperature may have determined a reduction in larval survival over the last decade (Figure S1).

### 3.6 Comparison with published cytb data

The available cytb sequences for Adriatic and Ionian samples (seven population samples from Maltagliati et al., 2010; Penant et al., 2013) provided a *F*_ST_ value of 0.02222, 10 times higher than the *F*_ST_ calculated for the SNPs dataset (*F*_ST_ = 0.00217). POWSIM analysis (Table 5) showed that the cytb *F*_ST_ value can be obtained, in the case of complete isolation, in as few as 2–5 generations assuming a population size of 100 females, or in a maximum of 100–120 generations when considering the highest female population size of 2,500 used in our forward‐time simulations. For all the combinations of generations and female population sizes, the power to detect differences with mtDNA was rather low (range 7.5%–39%), reflecting the small size of the population samples in the original study (*N* = 10 each). By contrast, the power of our panel of SNPs estimated by using the same number of generations and a fourfold greater diploid effective population size (as expected in the case of an equal sex ratio), was very high, approaching 100% in the majority of the cases. This result clearly indicates that the detection of differences between Adriatic–Ionian samples with cytb sequences but not with our panel of SNPs is very unlikely under the simulated conditions.

**Table 5:** Statistical power of SNPs in comparison with published cytb data. POWSIM simulations were performed on mitochondrial data using the cytb haplotype frequencies of 70 sequences from 7 Adriatic–Ionian populations (Santa Caterina di Nardò, Brindisi, Manfredonia, Lesina, Ancona, Mljet, Miramare) obtained originally in Maltagliati et al. (2010) and on nuclear SNPs using our eight Adriatic–Ionian samples. For cytb simulations, several values of female haploid effective population size (*N*_f_) were used in combination with a different number of generations of pure drift, to simulate a *F*_ST_ value of 0.0222, similar to that observed in Maltagliati et al. (2010) samples. Reported are: the *N*_f_ value used, the number of generations of drift (*t*), the average *F*_ST_ value obtained from 200 replicates and the power to detect differentiation, calculated as the proportion of significant tests at the end of the simulations using the Chi‐square test. For SNPs simulations, the same number of generations was tested using a diploid effective population size (*N*_e_) four times larger than the corresponding *N*_f_. The *N*_e_ values, number of generations of drift (*t*), average nuclear *F*_ST_ value obtained from 200 replicates and the power to detect differentiation, calculated as the proportion of significant tests at the end of the simulations using the Chi‐square test are reported

Cytb				SNPs			
*N*_f_	*t*	*F*_ST_	Power	*N*_e_	*t*	*F*_ST_	Power
100	2	0.0100	0.075	400	2	0.0025	1
	5	0.0249	0.385		5	0.0062	1
500	20	0.0198	0.245	2,000	20	0.0050	1
	25	0.0245	0.390		25	0.0062	1
1,000	40	0.0197	0.260	4,000	40	0.0050	1
	50	0.0250	0.380		50	0.0062	1
2,500	100	0.0198	0.195	10,000	100	0.0050	1
	120	0.0236	0.320		120	0.0069	1

## 4 Discussion

Overall, the 2b‐RAD population genomics analysis of the eight Adriatic–Ionian and two French‐Tunisian population samples of *Paracentrotus lividus* examined in this study provide clear support for genetic differentiation between the Central and Western Mediterranean Sea and for differentiation between samples collected along the French and Tunisian coasts. In the Central Mediterranean, genetic homogeneity was detected within and between Adriatic and Ionian basins, despite the use of more than 1,000 polymorphic loci. Lagrangian simulations in the Central Mediterranean (Adriatic and Ionian seas) predicted a potential larval exchange among locations, altogether supporting the connectivity of *P. lividus* in this area from a seascape genetics perspective.

### 4.1 Genetic differentiation at the large scale

Our results strongly support evidence of genetic partitioning among population samples collected throughout the entire study area, with a clear genetic break separating Central (Adriatic–Ionian) and Western (France–Tunisia) Mediterranean samples. This result is consistent with previous studies (Maltagliati et al., 2010; Penant et al., 2013), based mainly on mtDNA sequencing, where significant genetic divergence between the Adriatic Sea and the rest of the Mediterranean was detected. Notably, the pairwise *F*_ST_ values between Western and Central Mediterranean samples, although highly significant, were small, indicating that shallow differentiation exists also at this very wide geographic scale (>2,000 km).

In addition, the 2b‐RAD approach was able to detect a further, although weak, north‐to‐south differentiation in the Western Mediterranean, despite the relatively small sampling effort. This differentiation emerged both from pairwise comparisons and a hierarchical analysis of molecular variance supporting a subdivision of the samples into three geographic groups (Adriatic–Ionian basins, France, Tunisia) against any alternative partitioning including only two groups.

One of the most important advantages provided by genome‐wide approaches is the opportunity to disentangle the signal of differentiation obtained with neutral loci and with loci putatively under selection, potentially providing complementary information that could be used in defining conservation units (Funk, McKay, Hohenlohe, & Allendorf, 2012). The genomic scan analysis of sea urchin samples identified 17 putative outliers under directional selection. However, these loci were identified only by LOSITAN, which is very sensitive to deviations from a basic island model (Lotterhos & Whitlock, 2014; Whitlock & Lotterhos, 2015), whereas BAYESCAN did not detect outliers, raising the possibility that these outlier loci could simply be the product of genetic drift and a complex demographic history. Anyway, the inclusion of these 17 putative outliers did not change the general picture of differentiation. In fact, the exclusion of these loci resulted in smaller values of differentiation compared with the overall dataset but identified the same three geographic groups. The interpretation of these putative outliers as being truly under selection is premature, especially considering our limited sampling that prevented correlation of allelic frequencies with environmental variables. At present, it is worthwhile to note that nine of the 17 putative outliers matched with the recently released transcriptome of *P. lividus*, and six of them have a correspondence with *Strongylocentrotus purpuratus* proteins in the database of NCBI. The most intriguing match is for the fibrillary glycoprotein hyalin (Table S6), a key protein involved in the formation of the fertilization membrane, which avoids polyspermy just after fertilization (McClay & Fink, 1982). This match potentially adds a new gene linked to fertilization processes, in addition to the well‐known examples of gamete recognition proteins (Calderón, Turon, & Lessios, 2009; Pujolar & Pogson, 2011), for studies of sea urchins based on candidate genes.

### 4.2 Connectivity at the Adriatic–Ionian sea scale

Whether or not the putative outliers are taken into account, the results of our molecular analysis highlight an incontrovertible genetic homogeneity among the eight Adriatic–Ionian population samples. Both the analysis of molecular variance and all pairwise comparisons strongly support a pattern of genetic homogeneity in this area, where all the analyzed population samples seem to belong to a single panmictic population, at least from the genetic point of view. None of the pairwise *F*_ST_ comparisons was significant after correction for multiple tests, and only one pairwise comparison was below the uncorrected 0.05 probability threshold (TOG‐OTH).

Remarkably, SimuPOP forward‐time simulations provided clear indications that this Adriatic–Ionian genetic homogeneity is unlikely in the case of isolation, but is rather sustained by gene flow. In fact, in the case of isolation, the differentiation observed in the empirical dataset is reached and quickly exceeded in a few generations. On the other hand, forward‐time simulations with migration showed that few migrants (5–15 individuals depending on the population sizes) are sufficient to quickly and stably reach the *F*_ST_ value obtained for the Adriatic–Ionian basins. Taking into account the population sizes used in forward‐time simulations (Table S5), these small number of migrants correspond to a migration rate ranging from 0.15% to 5%, thus to a relatively high level of connectivity.

Notably, a scenario of genetic homogeneity with gene flow is consistent with the potential for larval connectivity obtained via Lagrangian simulations, performed by taking into account species‐specific life traits of spawning and dispersal. Simulations suggest that each sampling location within the Adriatic–Ionian basin is potentially connected to its closest neighbor (mainly in a counterclockwise direction, as explained above) through a relatively persistent flux of propagules. In addition to these along‐shore connections between neighboring locations, a few cross‐basin dispersal events (between the eastern and western coasts of the Adriatic) can take place in both directions and may sometimes reach also the furthest (Ionian) locations. In this regard, it is important to note that, according to our settings, the Lagrangian simulations should provide lower‐bound (conservative) estimates of potential connectivity. This conservativeness is the result of at least three major restrictions. First, we considered only a single spawning season between April and July (according to Sellem & Guillou, 2007), even though two possible spawning periods were reported for some location in the Mediterranean (spring; Byrne, 1990; Lozano et al., 1995; late summer–autumn; Régis, 1979; Fenaux et al., 1985; Pedrotti, 1993), and there is evidence for the presence of mature individuals all year round (Guettaf, San Martin, & Francour, 2000). Second, we included a water temperature effect in our model under the assumption that spawning occurs only below 18°C (Spirlet et al., 1998) and that larval survival is strongly reduced at temperatures above this threshold (Privitera et al., 2011). Both assumptions predict reduced connectivity during summer. Third, dispersal was modeled only among locations at which genetic sampling was performed, whereas rocky shore habitats suitable for *P. lividus* are widely available across the surveyed area. It is therefore very likely that stepwise, along‐shore connections between unsampled locations can greatly increase the potential for dispersal among the modeled locations. For all these reasons, we believe that our general picture of potential dispersal, suggesting a general mixing within the Adriatic–Ionian seas, provides support for the hypothesis that the genetic homogeneity observed in the area is due to exchange between locations.

### 4.3 Discrepancies with published cytb results

The absence of genetic structure within the Adriatic–Ionian basins obtained with SNPs is in sharp contrast with the significant differentiation detected in the same area using mtDNA sequences (Penant et al., 2013). Interestingly, in their study the authors obtained strong and significant differentiation for the Adriatic–Ionian population samples by reanalyzing published cytb data (Maltagliati et al., 2010) using pairwise *F*_ST_ values, and thus considering allelic frequency only. More generally, Penant et al. (2013) highlighted a much higher level of differentiation by using *F*_ST_ rather than taking into account genetic distances (Φ_ST_), with significant *F*_ST_ values among “nearly all population pairs within the Mediterranean and Adriatic basins, some of which were geographically close (about 40–60 km between our populations from the French Mediterranean coast)” and Φ_ST_ values capturing only differences at major transitions. This pattern was explained by a lack of informativeness of mtDNA mutations at the local scale, contrasted by the possibility to obtain significant *F*_ST_ values after relatively few generations of genetic drift (Penant et al., 2013). However, our POWSIM forward‐time simulations performed on cytb and SNPs data strongly point toward a more complex picture in the Adriatic and Ionian area. In fact, under a wide range of simulated divergence times and effective population sizes, and assuming a 1:1 sex ratio, significant *F*_ST_ values should be observed with our wide panel of SNPs loci but not with cytb, which is exactly the opposite of what we observed in our study.

It is important to note that these simulations rule out the possibility that an undetected unbalanced effective sex ratio can be at the basis of the different patterns observed with the two set of markers in the Adriatic–Ionian area, because the diploid effective population size itself is limited by a small number of effective females, being bound to an upper limit of four times the female population size in the case of a standard mating system (Wright, 1931).

Our forward‐time simulations showed the high power of our SNPs panel to detect differences in a few generations of isolation. For this reason, it is unlikely that mtDNA markers can detect differences that do not emerge from nuclear markers. Thus, a higher mutation rate and degree of polymorphisms of mtDNA markers compared to SNPs, and the uniparental inheritance and ploidy level, which make mtDNA more strongly affected by genetic drift (Ballard & Whitlock, 2004; Weersing & Toonen, 2009), are not likely explanations for the discrepancy between the mtDNA and SNPs results.

Alternatively, the contrasting pattern could be caused by selection or, perhaps, by the strongly skewed reproductive success of females. In this latter case, the successful reproduction of some females and the subsequent recruitment of their offspring would quickly increase maternal haplotype frequency leading to inflated differentiation. In particular, mtDNA markers can be influenced by differences in sex dispersal or by mating incompatibilities driven by females. The first case is not likely when fertilization is external and adults have limited mobility, as occurs in *P. lividus*. On the other hand, intraspecific mating incompatibilities between male/female pairs have been reported in sea urchins (Kregting, Thomas, Bass, & Yund, 2014); furthermore, in *P. lividus* differences in allelic frequency of bindin, a key gene involved in sperm recognition, were detected among otherwise genetically homogeneous cohorts (Calderón & Turon, 2010; Calderón, Palacín, et al., 2009).

### 4.4 Comparison with previous results in the same area and implications for MPAs

The geographic framework of this study is the selected Pilot Area of the FP7 CoCoNET project. The project aimed to investigate connectivity patterns of different species to understand the scale at which MPAs in this region can work as an effective network. Our findings support the connectivity in this area (at least for a species with a long PLD, as in the edible sea urchin). Our results are consistent with the evidence of complete homogeneity (obtained via both microsatellites and Lagrangian simulations) for the marbled crab *Pachygrapsus marmoratus*, a semiterrestrial crab inhabiting upper and middle tidal levels of rocky shores, with a PLD of 1 month, which was sampled approximately at the same locations (Marino et al., in review). Furthermore, our findings agree with the results obtained for the white sea bream (*Diplodus sargus sargus*), a relatively sedentary species (D'Anna, Giacalone, Pipitone, & Badalamenti, 2011; Di Franco et al., 2011) with a 1‐month PLD, for which an overall genetic homogeneity in the southwestern Adriatic Sea was observed using microsatellites (Di Franco et al., 2012; Pujolar et al., 2013). Based on this evidence, rocky shore populations of species with a high dispersal potential can be considered as effectively connected from the genetic point of view within the Adriatic–Ionian geographic region.

This suggests that a trans‐border approach (involving Italy, Croatia, Montenegro, Albania, and Greece) to the planning and management of MPA networks might be necessary. However, from a management perspective, it is essential to consider that the lack of a detectable genetic differentiation does not necessarily imply that *P. lividus* populations should be managed as a single unit, because the amount of migration needed to maintain genetically homogeneous populations is far smaller than the amount required to make them indistinguishable from a demographic viewpoint (Bortolotto, Bucklin, Mezzavilla, Zane, & Patarnello, 2011). Considering the threshold suggested by Waples and Gaggiotti (Waples & Gaggiotti, 2006), up to 10% of individuals can be exchanged at each generation among populations maintaining their demographic independence. In our simulations with migration (Table S5), the level of migration between populations (0.15%–5% per generation) was lower than 10% of individuals with any value of *N*_e_. Assuming that these values of *N*_e_ are sensible, the level of exchange between our samples is not high enough to justify their management as a single population. In contrast, taking into account the potential for self‐seeding suggested by the Lagrangian simulations (Tables 3 and 4), and the potential for larval export suggested by the genetic analyses, a local (yet coordinated) management of marine reserves may provide an effective conservation strategy that could also benefit the surrounding unprotected areas.

On the other hand, the comparison with other results recently produced in the context of the CoCoNET project (all referring to the same pilot study area) indicates that genetic homogeneity is not so common. For instance, while multispecies biophysical modeling (Melià et al., 2016) suggested a quite high potential connectivity for the black scorpionfish (*Scorpaena porcus*), a rocky shore fish characterized by a PLD of about 1 month, microsatellites genetic analyses pointed out a clear subdivision for the same species between the eastern and western part of the Adriatic and Ionian basins (Boissin et al., 2016). In addition, when considering a key ecological species, the habitat former *Posidonia oceanica*, whose propagules float passively for about 28 days, microsatellites showed a relatively low connectivity with a possible north‐to‐south partition (Jahnke et al., in review).

Although still based on a relatively small number of species, this comparison clearly warns against inferring a general pattern based only on the duration of the dispersal phase of each species. While a propagule duration of about 1 month may be sufficient to ensure genetic mixing in some species, such as *P. lividus*, it may be insufficient to produce genetic homogeneity in others. Specific dispersal traits of propagules (such as reproductive timing, depth range, behavioral features), together with differences in the spatial distribution, abundance and fecundity of their source populations, as well as recruitment success (further modulated by density‐dependent effects and ecological interactions), can indeed produce very different connectivity outcomes even when PLDs are very similar.

## 5 Conclusions and Future Lines of Research

Taken together, our results provide strong support for the existence of (i) genetic differentiation in *P. lividus* at the largest geographic scale within the Mediterranean Sea, and (ii) genetic mixing in the Adriatic–Ionian seas, where a smaller geographic scale and/or peculiar oceanographic features allow for an efficient exchange among locations for a species with long‐distance dispersal like *P. lividus*. However, comparison with the few published studies regarding connectivity conducted in the same area, even in species with a similar PLD, showed the existence of different patterns of genetic differentiation, ranging from complete panmixia to an east–west or north–south genetic partitioning. These findings warn against any generalization based on the presently available information and strongly indicate the need to obtain genetic connectivity data from a wider array of species, including keystone species such as habitat formers as well as rare or threatened species. At the same time, detailed biological data are needed to refine biophysical models, such as basic information about the correlation of physical and biological drivers of reproductive timing and larval mortality, which both have a strong influence on connectivity. The integration of all these aspects represents a crucial step toward providing a general framework of connectivity at the community level, and predicting expected changes in community structure in the future. It is also a crucial step toward the holistic perspective needed to achieve the Aichi Target 11 of the Convention on Biological Diversity, which aims at increasing the proportion of protected areas in the world, working as “a well‐connected system” by 2020 (Brooks, Dvorak, Spindler, & Miller, 2015).

## Data Accessibility

SNPs genotypes data together with the datasets supporting the conclusions of this article are available as supporting information and additional files in DRYAD at doi:10.5061/dryad.73kc6. Short read data are deposited in SRA with accessions SRR5266202‐SRR5266474.

## Conflict of Interest

None declared.

## Author Contributions

MP, PM, MS, and LZ conceived and designed the experiments and wrote the manuscript. MP and MS performed the experiments. MP, PM, MS, and LZ analyzed the data. PM, LZ, and TP contributed reagents/materials/analysis tools. GA, JBS, EB, RC, AC, MC, LC, GG, CK, VM, IAMM, PM, CP, TP, MP, MS, and LZ critically reviewed the manuscript; the final version of the manuscript was approved by all the authors.
