# Supplementary material for: Population genomics meet Lagrangian simulations: Oceanographic patterns and long larval duration ensure connectivity among Paracentrotus lividus populations in the Adriatic and Ionian seas
Source: Ecol Evol. 2017 Mar 14;7(8):2463–79. doi: 10.1002/ece3.2844 (PMC5395429; doi:10.1002/ece3.2844)
Supplement: Supplementary file 1 [file ECE3-7-2463-s001.docx]

Table S1. 2b-RAD oligonucleotide sequences used for Illumina next-generation sequencing.

| ID | Oligo | Sequences |
| --- | --- | --- |
| ADPT1 ss | Anti-ILL | AGATCGGAAGAGC/3InvdT |
| ADAPT2 ss | 5ILL-NN | CTACACGACGCTCTTCCGATCTNN |
| ADAPT3 ss | 3ILL-NN | CAGACGTGTGCTCTTCCGATCTNN |
| Primer F | ILL-Mpx | AATGATACGGCGACCACCGAGATCTACACTCTTTCCCTACACGACGCTCTTCCGAT |
| Barcoded-primer R | ILL-RAD-bc | CAAGCAGAAGACGGCATACGAGAT [barcode] GTGACTGGAGTTCAGACGTGTGCTCTTCCGAT |
| 2bRAD amp F | IC1P5 | AATGATACGGCGACCACCGA |
| 2bRAD amp R | IC2-P7 | CAAGCAGAAGACGGCATACGA |

Table S2. Validated loci by PCR based-approach and Sanger sequencing. Locus ID, consensus sequence, primer sequences (Forward and Reverse) and polymorphism level for each locus, sample ID and predicted genotypes for each sample are reported. SNP position is highlighted in bold (consensus sequences).

| Locus ID | Consensus sequence | Primer sequences (5’-3’) | Polymorphism level | Samples ID | Genotypes |
| --- | --- | --- | --- | --- | --- |
| 1110 | TGACTGTGTACAAGGATCGTGGC**G**TTGCACTG | For – CCATCTTGTCCCGAAGATGTC  Rev - AGCAACCATCGGTTCCAGGAT | 1 SNP | PL_CZ2 | G |
|  |  |  |  | PL_CZ12 | G |
|  |  |  |  | PL_MN26 | G |
|  |  |  |  | PL_CZ5 | G/A |
|  |  |  |  | PL_TG12 | G/A |
|  |  |  |  | PL_TG15 | G/A |
| 90 | CTGGACCAATCAATGATGGTGGA**G**GGT**G**CATC | For - AAAACGATACCACCATGACCTC  Rev - AAGGATAGTCTTTGAAACCGGTC | 2 SNPs | PL_TG27 | GG |
|  |  |  |  | PL_OT9 | GG |
|  |  |  |  | PL_TR33 | GG/TG |
|  |  |  |  | PL_TG20 | GG/GA |
|  |  |  |  | PL_OT7 | GG/TG |
|  |  |  |  | PL_CZ5 | GG/TG |
| 3987 | CCTG**C**AGCAGCAAAGCTTGTGG**A**TACATGAAG | For - GATACCCCGCCAAAGTTGAG  Rev - GGTGAACTCCTGCACTTGAT | 2 SNPs | PL_MN6 | CA |
|  |  |  |  | PL_GR26 | CA |
|  |  |  |  | PL_CZ30 | CA/TA |
|  |  |  |  | PL_PC24 | CA/AA |
|  |  |  |  | PL_ALB16 | CA/CC |
|  |  |  |  | PL_TG8 | CA/CC |
| 6408 | CTCCCCCACCCAATCCCAGTGGTGACAACATA | For - GCAGTAAGGGGTCGTCCACAA  Rev - GGCGTGTGAGCAGGTTGAGACC | 0 SNP (monomorphic) | PL_CZ12 | Consensus seq |
|  |  |  |  | PL_OT23 | Consensus seq |
|  |  |  |  | PL_OT26 | Consensus seq |
|  |  |  |  | PL_MN7 | Consensus seq |

Table S3. Genetic variability within *Paracentrotus lividus* population samples examined in the study. The genetic variability was assessed on the total dataset (1122 polymorphic loci) and on the dataset filtered for a minor allele frequency (MAF) of 0.01 (494 polymorphic loci, asterisk). For each population sample, observed heterozygosity (H_O_), unbiased expected heterozygosity (H_E_) and allelic richness (A_R_) are reported; allelic richness is calculated with a minimum sample size of 16 diploid individuals.

| Acronym | H_O_ | H_E_ | A_R_ | *H_O_ | *H_E_ | *A_R_ |
| --- | --- | --- | --- | --- | --- | --- |
| OTH | 0.0699 | 0.0750 | 1.28 | 0.1509 | 0.1611 | 1.56 |
| KAP | 0.0709 | 0.0766 | 1.28 | 0.1523 | 0.1638 | 1.57 |
| BOK | 0.0651 | 0.0742 | 1.27 | 0.1423 | 0.1616 | 1.56 |
| KOR | 0.0655 | 0.0728 | 1.27 | 0.1424 | 0.1587 | 1.56 |
| TRE | 0.0680 | 0.0752 | 1.28 | 0.1455 | 0.1603 | 1.55 |
| TOG | 0.0679 | 0.0756 | 1.28 | 0.1446 | 0.1612 | 1.56 |
| OTR | 0.0682 | 0.0774 | 1.29 | 0.1455 | 0.1648 | 1.57 |
| POC | 0.0697 | 0.0769 | 1.28 | 0.1512 | 0.1669 | 1.58 |
| FRN | 0.0731 | 0.0775 | 1.30 | 0.1566 | 0.1654 | 1.59 |
| TUN | 0.0705 | 0.0802 | 1.30 | 0.1495 | 0.1698 | 1.59 |

Table S4. Estimates of Adriatic-Ionian effective population sizes based on the Linkage Disequilibrium method by NeEstimator. Given the genetic homogeneity found in the Adriatic-Ionian basins, the estimates were calculated by pooling all the Adriatic-Ionian samples. Three allele frequencies thresholds are used and, for each one, the corresponding Jackknife 95% confidence interval is reported.

|  | **LOWEST ALLELE FREQUENCY** | | |
| --- | --- | --- | --- |
|  | **0.05** | **0.02** | **0.01** |
| **Jackknife 95% CIs** | 216.8 – 290.2 | 452.4 – 609.3 | 723.9 – 1009.1 |

Table S5. Forward-time simulations of alternative scenarios of isolation and migration obtained with SimuPOP for the 8 Adriatic-Ionian population samples. Two alternative scenarios of isolation and migration were tested: first, a pure drift model where the populations were allowed to diverge for a fixed number of generations with the number of migrants/generation (Nm) set to 0; then, a scenario of migration where, for each predefined population size, the exact number of migrants between populations per generation needed to consistently achieve the observed F_ST_ was recorded. Specifically, 8 ideal populations were simulated starting from the Adriatic-Ionian SNP frequencies; the sex ratio was assumed to be 1:1 and a random mating scheme was allowed; at each generation, parents died after the reproduction event and their offspring received the alleles by Mendelian inheritance. Different population sizes were used (100 – 10 000); this interval was chosen so as to include a small value of 100 individuals, corresponding to the smallest effective population size (Ne) estimated from *P. lividus* microsatellites temporal variation (Calderón *et al.* 2009a), values corresponding to new effective population sizes estimated with the Linkage Disequilibrium method by NeEstimator (Do *et al.* 2014; Table S4), and few arbitrarily high values. All the simulations were conducted with a universal standard mutation rate (2.5 × 10^-8^; Pontes *et al.* 2015) and a faster mutation rate (1 × 10^-6^). For simulations under isolation, the table reports the tested population sizes (N), the number of generations of divergence (t_d_) needed to reach and exceed the observed *F*_ST_ in the empirical dataset (0.00217) and the final F_ST_ obtained. For simulations with migration, N is reported along with the number of generations (t_eq_) and the number of migrants per generation (Nm) needed to stably achieve the observed *F*_ST_.

| **ISOLATION** | | | **MIGRATION** | | |
| --- | --- | --- | --- | --- | --- |
| **N** | **t_d_** | **Final F_ST_** | **N** | **t_eq_** | **Nm** |
| **100** | 1 | 0.00520 | **100** | 2 | 5 |
| **400** | 2 | 0.00249 | **400** | 8 | 10 |
| **1000** | 5 | 0.00247 | **1000** | 15 | 12 |
| **2000** | 9 | 0.00223 | **2000** | 30 | 13 |
| **4000** | 18 | 0.00218 | **4000** | 60 | 14 |
| **10 000** | 45 | 0.00222 | **10 000** | 150 | 15 |

Table S6. Putative outliers under positive selection identified with LOSITAN by comparing Central (Adriatic-Ionian) and Western (France and Tunisia) population samples. Locus ID, consensus sequence, contig ID of *Paracentrotus lividus* transcriptome (NCBI database, accession number GCZS00000000.1; Gildor *et al.* 2016) and *Strongylocentrus purpuratus* proteins providing the top hit and related NCBI accession number are reported. The position of the SNP with the highest expected heterozygosity (consensus sequences) is underlined and bold. Asterisks indicate outlier loci displayed a match with more than one contig, for convenience only one contig ID is reported since they were identical.

| Locus ID | Consensus sequences | *P. lividus* contig ID | *S. purpuratus* protein |
| --- | --- | --- | --- |
| 342 | TACCAACTTTCAAGCAAGGTGGACACA**A**ACCC | gb\|GCZS01021365.1\| | TBC1 domain family member 1 (LOC100891782) |
| 16413 * | TAAATGGAAGCAAGGAATGTGGTCAAGTGA**C**G | gb\|GCZS01088153.1\| | cyclin-D-binding Myb-like transcription factor 1 (LOC754814) |
| 1230 * | AGCAGTTATTCAAACCGGGTGGGAAGGA**C**GAA | gb\|GCZS01098438.1\| | mannosyl-oligosaccharide alpha-1,2-mannosidase isoform B (LOC581367) |
| 3050 | A**C**ATCCCCTTCAATGTACGTGGATTTCCAGTG | - | - |
| 10028 | GCACTCACTGCAAAGTGCGTGGTCATTCA**G**AA | gb\|GCZS01138538.1\| | myosin VII (LOC373219) |
| 12431 | TGTGAGAGTACAATCTTTGTGGT**A**GGCTGAAA | - | - |
| 20795 * | CCATACTCATCAAATCG**G**GTGGGATCAGTCCT | gb\|GCZS01130689.1\| | - |
| 21392 | TCACCTGACCCAAACCTAGTGGTAG**G**TATCAT | - | - |
| 22169 * | CTGTTCAGCTCAAGGA**C**GGTGGTATGGCTTCT | gb\|GCZS01075885.1\| | - |
| 23920 | GAGAATGATACAATCAGAGTGGCAAAGC**T**GTC | - | - |
| 26862 | CCAGAGACATCAAAGGCTGTGGGTTC**T**GCAAA | gb\|GCZS01121683.1\| | hyalin (LOC373362) |
| 27918 | ACATAATGCTCAACTTCCGTGGTCACGA**T**AAT | - | - |
| 4345 * | GGTCTAAGGCCAATG**C**AGGTGGTACACCCATC | gb\|GCZS01108283.1\| | - |
| 17408 * | TGGATCATGTCAATATTTGTGGC**A**CTTTCCTG | gb\|GCZS01108741.1\| | cystine/glutamate transporter (LOC584098) |
| 19300 | CAACATGGTGCAACACGAGTGGTTT**C**GATCAT | - | - |
| 23162 | CTGTGCATCTCAAGTAAGGTGGCA**C**TAATTAG | - | - |
| 23417 | GGGTATAAAACAAGCAATGTGGCCTTTGC**C**CA | - | - |

Table S7. Pairwise genetic distances (*F*_ST_) between Central (Adriatic-Ionian) and Western (France and Tunisia) population samples based on 1105 neutral loci. Benjamini & Hochberg correction for multiple tests was applied. *F*_ST_ indices and p-values are reported below and above the diagonal, respectively; significant indices and p-values in bold. Comparisons between Adriatic-Ionian populations are shaded in grey. See Table 1 in the main text for location acronyms.

|  | OTH | KAP | BOK | KOR | TRE | TOG | OTR | POC | FRN | TUN |
| --- | --- | --- | --- | --- | --- | --- | --- | --- | --- | --- |
| OTH |  | 0.2520 | 0.3405 | 0.7206 | 0.3305 | 0.0285 | 0.0555 | 0.4877 | **<0.0001** | **<0.0001** |
| KAP | 0.00242 |  | 0.0658 | 0.8763 | 0.5159 | 0.8086 | 0.4628 | 0.4101 | **0.0133** | **<0.0001** |
| BOK | 0.00278 | 0.00515 |  | 0.1076 | 0.3026 | 0.4549 | 0.2575 | 0.0560 | **<0.0001** | **0.0002** |
| KOR | 0.00063 | -0.00031 | 0.00544 |  | 0.8254 | 0.4738 | 0.8065 | 0.5255 | **0.0001** | **0.0001** |
| TRE | 0.00239 | 0.00154 | 0.00338 | 0.00027 |  | 0.2924 | 0.5426 | 0.9472 | **<0.0001** | **0.0023** |
| TOG | 0.00525 | 0.00024 | 0.00269 | 0.00229 | 0.00297 |  | 0.7601 | 0.8072 | **0.0002** | **<0.0001** |
| OTR | 0.00477 | 0.00202 | 0.00395 | 0.00071 | 0.00192 | 0.00095 |  | 0.6076 | **<0.0001** | **0.0001** |
| POC | 0.00164 | 0.00198 | 0.00581 | 0.00194 | -0.00088 | 0.00049 | 0.00161 |  | **<0.0001** | **<0.0001** |
| FRN | **0.01203** | **0.00553** | **0.01655** | **0.01026** | **0.01056** | **0.00824** | **0.01391** | **0.00964** |  | **0.0120** |
| TUN | **0.02048** | **0.01543** | **0.01525** | **0.01452** | **0.01110** | **0.01818** | **0.01418** | **0.01668** | **0.00835** |  |


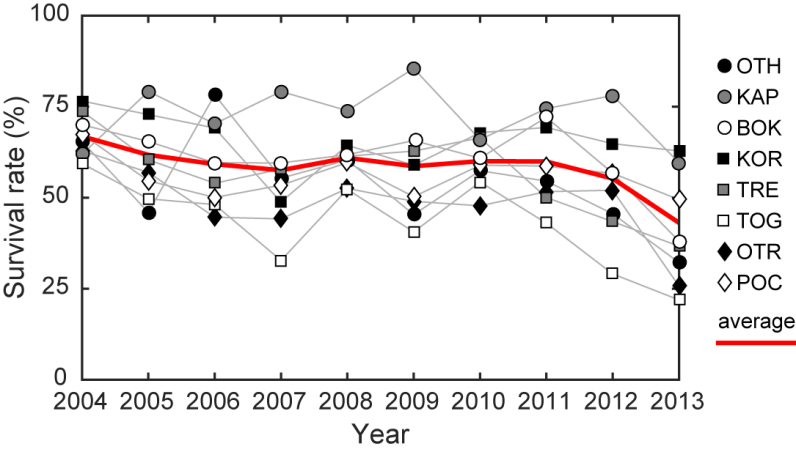


Figure S1. Survival rate trend 2004–2013. Survival rate is defined as the proportion of particles surviving their pelagic larval phase (independent of the actual success of migration from the location of origin to that of the destination). Symbols (connected by thin grey lines) indicate release locations (see Table 1 for acronyms), while the red line represents the yearly average across locations.
